# Ethyl 1,3,10,12-tetra­phenyl-19,20-dioxa­hexa­cyclo­[10.6.1.1^3,10^.0^2,11^.0^4,9^.0^13,18^]icosa-4(9),5,7,13(18),14,16-hexa­ene-2-carboxyl­ate

**DOI:** 10.1107/S1600536810045873

**Published:** 2010-11-13

**Authors:** P. Narayanan, K. Sethusankar, Meganathan Nandhakumar, Arasambattu K. Mohanakrishnan

**Affiliations:** a Department of Physics, RKM Vivekananda College (Autonomous), Chennai 600 004, India; b Department of Organic Chemistry, University of Madras, Guindy Campus, Chennai 600 025, India

## Abstract

The title compound, C_45_H_34_O_4_, is the product of a tandem ‘pincer’ Diels–Alder reaction consisting of two consecutive [4 + 2] cyclo­additions between two 2-benzofuran units and ethyl propiolate. The mol­ecule comprises a fused hexa­cyclic system containing four five-membered rings, which are in the usual envelope conformation, and two six-membered rings. In addition, four phenyl rings are attached to the hexa­cyclic system. The packing is stabilized by C—H⋯π inter­actions.

## Related literature

For the tandem ‘pincer’ Diels–Alder reaction, see: Lautens & Fillion (1997 ▶). For related structures, see: Gurbanov *et al.* (2009 ▶); Toze *et al.* (2010 ▶).
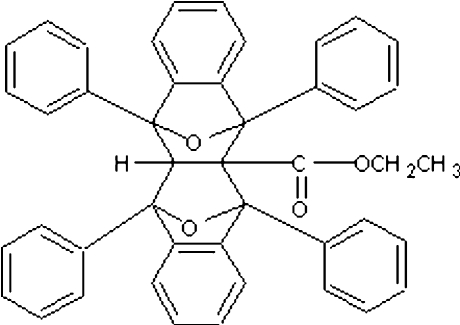


## Experimental

### 

#### Crystal data

C_45_H_34_O_4_
*M* *_r_* = 638.72
Orthorhombic, 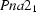

*a* = 17.2498 (4) Å
*b* = 12.5137 (3) Å
*c* = 15.4118 (5) Å
*V* = 3326.77 (15) Å^3^
*Z* = 4
Mo *K*α radiation
μ = 0.08 mm^−1^
*T* = 295 K
0.30 × 0.20 × 0.20 mm

#### Data collection

Bruker Kappa APEXII CCD diffractometer
Absorption correction: multi-scan (*SADABS*; Sheldrick, 1996 ▶) *T*_min_ = 0.962, *T*_max_ = 0.989
19378 measured reflections
5474 independent reflections
4366 reflections with *I* > 2σ(*I*)
*R*_int_ = 0.041

#### Refinement

*R*[*F* ^2^ > 2σ(*F* ^2^)] = 0.038
*wR*(*F* ^2^) = 0.092
*S* = 1.00
5474 reflections
443 parameters
1 restraint
H-atom parameters constrained
Δρ_max_ = 0.16 e Å^−3^
Δρ_min_ = −0.17 e Å^−3^

### 

Data collection: *APEX2* (Bruker, 2004 ▶); cell refinement: *SAINT* (Bruker, 2004 ▶); data reduction: *SAINT*; program(s) used to solve structure: *SHELXS97* (Sheldrick, 2008 ▶); program(s) used to refine structure: *SHELXL97* (Sheldrick, 2008 ▶); molecular graphics: *ORTEP-3* (Farrugia, 1997 ▶); software used to prepare material for publication: *SHELXL97* and *PLATON* (Spek, 2009 ▶).

## Supplementary Material

Crystal structure: contains datablocks global, I. DOI: 10.1107/S1600536810045873/rk2244sup1.cif

Structure factors: contains datablocks I. DOI: 10.1107/S1600536810045873/rk2244Isup2.hkl

Additional supplementary materials:  crystallographic information; 3D view; checkCIF report

## Figures and Tables

**Table 1:** Hydrogen-bond geometry (Å, °) *Cg*8 and *Cg*10 are the centroids of the C15–C20 and C28–C33 rings, respectively.

*D*—H⋯*A*	*D*—H	H⋯*A*	*D*⋯*A*	*D*—H⋯*A*
C4—H4⋯*Cg*10^i^	0.93	2.97	3.740 (3)	141
C35—H35⋯*Cg*8	0.93	2.60	3.446 (2)	151
C44—H44⋯*Cg*8^ii^	0.93	2.87	3.671 (3)	145

Symmetry codes: (i) 
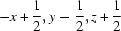
; (ii) 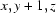.

**Fig. 1.**
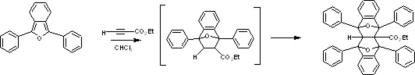
Construction of the tandem "pincer" Diels-Alder adduct of ethyl propiolate.

**Fig. 2.**
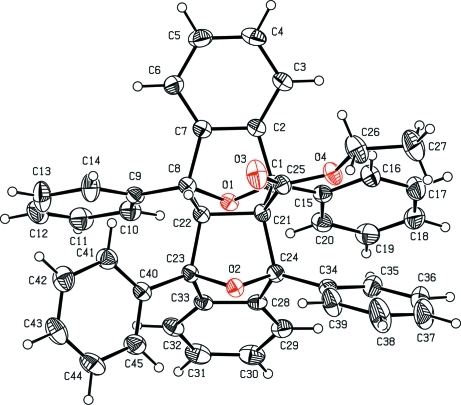
The molecular structure of the title compound with the atom numbering scheme, Displacement ellipsoids are drawn at 30° probability level. H atoms are present as a small spheres of arbitrary radius.

## supplementary crystallographic information

### Comment

The tandem "pincer" Diels–Alder reaction, consisting of two consecutive [4+2] cycloadditions between two dienes and an acetylenic bis–dienophile when furan derivatives are used as the diene components. Where, in our case two benzo(C)furans and ethyl propiolate are used as the diene components and acetylenic bis–dienophile components, respectively as shown in (Fig. 1). (Lautens & Fillion, 1997).

The title compound C_45_H_34_O_4_, comprises a fused hexacyclic system and four phenyl rings attached with this system. The hexacyclic system consists of four 5–membered rings and two phenyl rings. In addition to that, two phenyl rings at the top and bottom of the system and also four phenyl rings are attached on the bothside of the system. The X–ray analysis confirms the molecular structure and atom connectivity as illustrated in (Fig. 2). All four 5–membered rings are in the usual 'envelope' conformation.

The two 6–membered rings C1/C2/C7/C8/C22/C21 and C24/C21/C22/C23/C33/C28 are nearly coplanar having a dihedral angle of 2.76 (12)°. The dihedral angle between the rings C1/C2/C7/C8/O1 and C1/C21/C22/C8/O1; C24/C21/C22/C23/O2 and C24/C28/C33/C23/O2 are 82.87 (13)°; 86.80 (13)°, respectively.

In the 5–membered ring C1/C2/C7/C8/O1, the deviation of atom O1 is -0.3206 (16)Å, the puckering parameters of the ring are Q_2_ = 0.5070 (19)Å and φ_2_ = 180.2 (2)°. This ring adopts the 'envelope' conformation on O1. In the 5–membered ring C1/C21/C22/C8/O1, the deviation of atom O1 is 0.3496 (16)Å. the puckering parameters are Q_2_ = 0.5530 (19)Å and φ_2_ = 359.5 (2)°. This ring adopts 'envelope' conformation on O1.

In the five membered ring C24/C21/C22/C23/O2, the deviation of atom O2 is -0.3734 (15)Å, the puckering parameters of the ring are Q_2_ = 0.5914 (19)Å and φ_2_ = 176.6 (2)°. This ring adopts the envelope conformation on O2. In the five membered ring C24/C28/C33/C23/O2, the deviation of atom O2 is 0.3297 (15)Å. The puckering parameters are Q_2_ = 0.5216 (19)Å and φ_2_ = 0.2 (2)°. This ring adopts 'envelope' conformation on O2.

In the six membered ring C1/C2/C7/C8/C22/C21, the deviation of atoms C1 and C8 are -0.581 (2)Å, -0.573 (2)Å respectively.the puckering parameters of the ring are Q_2_=1.000 (2)Å and φ_2_=178.94 (12)°.This ring adopts the Boat conformation(B-form). In the six membered ring C24/C21/C22/C23/C33/C28, the deviation of atom C23 and C24 are -0.541 (2)Å, -0.536 (2)Å respectively. The puckering parameters of the ring are Q_2_=0.933 (2)Å and φ_2_=118.83 (13)°. This ring adopts the 'boat' conformation(B–form).

The molecular structure is stabilized by C—H···*Cg* interactions - look Table 1, where *Cg*8 is center of gravity C15/C16/C17/C18/C19/C20 ring and *Cg*10 is center of gravity C28/C29/C30/C31/C32/C33 ring. Symmetry codes: (i) -*x*+1/2, *y*-1/2, *z*+1/2; (ii) *x*, *y*+1, *z*.

### Experimental

To a solution of benzo[*c*]furan (0.5 g, 1.85 mmol) in dry CHCl_3_ (20 ml), ethyl propiolate (0.20 g, 2.04 mmol) was added and the reaction mixture was stirred for 2 h at reflux under nitrogen atmosphere. The solvent was removed *in vacuo* to give crude compound which on washing with ethanol gave adduct as a colourless solid. See the (Fig. 1). Yield: 0.53 g (45%). M.P.: 451–453 K. IR (KBr): 1719, 1600, 1505, 755 cm^-1^.

^1^H NMR (300 MHz, CDCl_3_): δ 7.89–7.80 (2*H*, m), 7.65–7.60 (2*H*, m), 7.55–7.50 (5*H*, m), 7.49–7.44 (5*H*, m), 7.09–7.06 (7*H*, m), 6.98–6.95 (7*H*,m), 4.69 (1*H*, s), 3.76–3.44 (2*H*, m), 0.90 (3*H*, t, J = 7.2 Hz);

^13^C NMR (75 MHz, CDCl_3_): δ 170.7, 149.9, 147.0, 144.9, 144.8, 137.9, 136.3, 134.6, 134.5, 129.6, 128.8, 128.3, 128.2, 128.1, 127.8, 127.6, 127.5, 127.4, 127.2, 127.1, 127.0, 126.6, 126.5, 126.3, 125.6, 123.0, 122.6, 121.3, 119.0, 90.9, 88.6, 88.3,86.7, 78.7, 67.3, 61.1, 13.6.

### Refinement

The hydrogen atoms were placed in calculated positions with C—H = 0.93Å to 0.98Å and refined in the riding model with fixed isotropic displacement parameters: *U*_iso_(H) = 1.5*U*_eq_(C) for CH_3_ groups and *U*_iso_(H) = 1.2*U*_eq_(C) for the other groups.

In the diffraction experiment were measured 1750 Friedel pairs. Because no heavy atoms (*Z* > Si) in molecule, during refinement by *SHELXL97*, was used 'MERG 2' instruction and in final CIF descriptors were placed: _refine_ls_abs_structure_Flack "?" and _chemical_absolute_configuration "unk" (Flack, 1983).

### Crystal data

**Table:**

C_45_H_34_O_4_	*D*_x_ = 1.275 Mg m^−^^3^
*M**_r_* = 638.72	Melting point = 451–453 K
Orthorhombic, *P**n**a*2_1_	Mo *K*α radiation, λ = 0.71073 Å
Hall symbol: P 2c -2n	Cell parameters from 5474 reflections
*a* = 17.2498 (4) Å	θ = 1.0–26.9°
*b* = 12.5137 (3) Å	µ = 0.08 mm^−^^1^
*c* = 15.4118 (5) Å	*T* = 295 K
*V* = 3326.77 (15) Å^3^	Block, colourless
*Z* = 4	0.30 × 0.20 × 0.20 mm
*F*(000) = 1344	

### Data collection

**Table:**

Bruker Kappa APEXII CCD diffractometer	5474 independent reflections
Radiation source: fine–focus sealed tube	4366 reflections with *I* > 2σ(*I*)
graphite	*R*_int_ = 0.041
ω scans	θ_max_ = 26.9°, θ_min_ = 2.4°
Absorption correction: multi-scan (*SADABS*; Sheldrick, 1996)	*h* = −21→20
*T*_min_ = 0.962, *T*_max_ = 0.989	*k* = −15→15
19378 measured reflections	*l* = −14→19

### Refinement

**Table:**

Refinement on *F*^2^	Primary atom site location: structure-invariant direct methods
Least-squares matrix: full	Secondary atom site location: difference Fourier map
*R*[*F*^2^ > 2σ(*F*^2^)] = 0.038	Hydrogen site location: inferred from neighbouring sites
*wR*(*F*^2^) = 0.092	H-atom parameters constrained
*S* = 1.00	*w* = 1/[σ^2^(*F*_o_^2^) + (0.0491*P*)^2^ + 0.1034*P*] where *P* = (*F*_o_^2^ + 2*F*_c_^2^)/3
5474 reflections	(Δ/σ)_max_ < 0.001
443 parameters	Δρ_max_ = 0.16 e Å^−^^3^
1 restraint	Δρ_min_ = −0.16 e Å^−^^3^

### Special details

**Table:**

Geometry. All s.u.'s (except the s.u. in the dihedral angle between two l.s. planes) are estimated using the full covariance matrix. The cell s.u.'s are taken into account individually in the estimation of s.u.'s in distances, angles and torsion angles; correlations between s.u.'s in cell parameters are only used when they are defined by crystal symmetry. An approximate (isotropic) treatment of cell s.u.'s is used for estimating s.u.'s involving l.s. planes.
Refinement. Refinement of *F*^2^ against ALL reflections. The weighted *R*–factor *wR* and goodness of fit *S* are based on *F*^2^, conventional *R*–factors *R* are based on *F*, with *F* set to zero for negative *F*^2^. The threshold expression of *F*^2^ > σ(*F*^2^) is used only for calculating *R*–factors(gt) *etc*. and is not relevant to the choice of reflections for refinement. *R*–factors based on *F*^2^ are statistically about twice as large as those based on *F*, and *R*–factors based on ALL data will be even larger.

### Fractional atomic coordinates and isotropic or equivalent isotropic displacement parameters (Å^2^)

**Table:**

	*x*	*y*	*z*	*U*_iso_*/*U*_eq_	
C1	0.20543 (10)	0.38628 (15)	0.29734 (15)	0.0333 (4)	
C2	0.19096 (11)	0.35324 (15)	0.39052 (15)	0.0349 (4)	
C3	0.21738 (13)	0.27151 (17)	0.44218 (17)	0.0440 (5)	
H3	0.2527	0.2216	0.4211	0.053*	
C4	0.18998 (15)	0.2655 (2)	0.52647 (19)	0.0574 (7)	
H4	0.2064	0.2100	0.5621	0.069*	
C5	0.13896 (15)	0.3400 (2)	0.55862 (19)	0.0571 (6)	
H5	0.1212	0.3338	0.6154	0.069*	
C6	0.11365 (12)	0.42444 (18)	0.50730 (17)	0.0442 (5)	
H6	0.0805	0.4764	0.5293	0.053*	
C7	0.13913 (11)	0.42854 (15)	0.42326 (15)	0.0356 (5)	
C8	0.12338 (10)	0.50524 (14)	0.34842 (15)	0.0331 (4)	
C9	0.04568 (10)	0.55950 (15)	0.34911 (16)	0.0375 (5)	
C10	−0.01251 (11)	0.52952 (19)	0.29307 (18)	0.0487 (6)	
H10	−0.0041	0.4746	0.2535	0.058*	
C11	−0.08388 (13)	0.5812 (2)	0.2956 (2)	0.0619 (7)	
H11	−0.1231	0.5603	0.2578	0.074*	
C12	−0.09681 (14)	0.6620 (2)	0.3526 (2)	0.0659 (8)	
H12	−0.1442	0.6974	0.3530	0.079*	
C13	−0.04019 (15)	0.6907 (2)	0.4090 (3)	0.0740 (10)	
H13	−0.0491	0.7454	0.4486	0.089*	
C14	0.03077 (13)	0.63902 (19)	0.4079 (2)	0.0594 (7)	
H14	0.0688	0.6586	0.4476	0.071*	
C15	0.22514 (12)	0.30145 (15)	0.23155 (15)	0.0372 (5)	
C16	0.29011 (13)	0.23665 (16)	0.24033 (19)	0.0489 (6)	
H16	0.3211	0.2423	0.2895	0.059*	
C17	0.30888 (15)	0.16377 (18)	0.1762 (2)	0.0585 (7)	
H17	0.3528	0.1214	0.1825	0.070*	
C18	0.26396 (16)	0.15304 (19)	0.1038 (2)	0.0595 (7)	
H18	0.2780	0.1053	0.0603	0.071*	
C19	0.19801 (15)	0.21312 (18)	0.09574 (19)	0.0564 (7)	
H19	0.1662	0.2044	0.0476	0.068*	
C20	0.17865 (14)	0.28670 (16)	0.15910 (17)	0.0456 (5)	
H20	0.1337	0.3270	0.1530	0.055*	
C21	0.25748 (10)	0.49143 (14)	0.30407 (15)	0.0319 (4)	
C22	0.19772 (10)	0.57437 (14)	0.33934 (15)	0.0313 (4)	
H22	0.2141	0.6002	0.3965	0.038*	
C23	0.20355 (10)	0.66599 (14)	0.27165 (14)	0.0329 (4)	
C24	0.28491 (11)	0.54884 (15)	0.21709 (15)	0.0350 (5)	
C25	0.32570 (11)	0.48322 (16)	0.36640 (16)	0.0381 (5)	
C26	0.44065 (14)	0.3879 (2)	0.4005 (2)	0.0689 (8)	
H26A	0.4268	0.3728	0.4602	0.083*	
H26B	0.4708	0.4534	0.3992	0.083*	
C27	0.48667 (16)	0.2985 (2)	0.3642 (3)	0.0871 (11)	
H27A	0.4561	0.2344	0.3648	0.131*	
H27B	0.5324	0.2882	0.3986	0.131*	
H27C	0.5013	0.3150	0.3056	0.131*	
C28	0.21839 (11)	0.55562 (14)	0.15257 (15)	0.0345 (4)	
C29	0.20378 (13)	0.51152 (17)	0.07231 (17)	0.0451 (5)	
H29	0.2372	0.4611	0.0486	0.054*	
C30	0.13833 (16)	0.54386 (19)	0.02773 (18)	0.0552 (6)	
H30	0.1285	0.5164	−0.0273	0.066*	
C31	0.08712 (14)	0.61661 (19)	0.06384 (19)	0.0543 (7)	
H31	0.0430	0.6367	0.0332	0.065*	
C32	0.10079 (12)	0.65951 (16)	0.14468 (17)	0.0431 (5)	
H32	0.0659	0.7071	0.1697	0.052*	
C33	0.16744 (11)	0.63003 (14)	0.18725 (15)	0.0343 (5)	
C34	0.36412 (12)	0.52056 (16)	0.18220 (16)	0.0406 (5)	
C35	0.37622 (13)	0.42842 (18)	0.13515 (18)	0.0508 (6)	
H35	0.3347	0.3831	0.1236	0.061*	
C36	0.44885 (15)	0.4026 (2)	0.1051 (2)	0.0671 (8)	
H36	0.4563	0.3394	0.0744	0.080*	
C37	0.51000 (16)	0.4690 (2)	0.1199 (3)	0.0912 (13)	
H37	0.5589	0.4525	0.0983	0.109*	
C38	0.49869 (14)	0.5601 (2)	0.1669 (3)	0.0967 (15)	
H38	0.5405	0.6051	0.1777	0.116*	
C39	0.42635 (13)	0.58658 (18)	0.1986 (2)	0.0661 (9)	
H39	0.4196	0.6487	0.2308	0.079*	
C40	0.18919 (10)	0.78078 (15)	0.29744 (16)	0.0359 (5)	
C41	0.17398 (14)	0.81397 (17)	0.38066 (18)	0.0511 (6)	
H41	0.1721	0.7642	0.4254	0.061*	
C42	0.16133 (16)	0.92160 (19)	0.3985 (2)	0.0619 (7)	
H42	0.1492	0.9428	0.4547	0.074*	
C43	0.16647 (15)	0.99565 (18)	0.3347 (2)	0.0625 (8)	
H43	0.1578	1.0674	0.3469	0.075*	
C44	0.18442 (15)	0.96424 (18)	0.2525 (2)	0.0646 (8)	
H44	0.1895	1.0151	0.2089	0.078*	
C45	0.19522 (13)	0.85747 (16)	0.23348 (19)	0.0513 (6)	
H45	0.2067	0.8370	0.1769	0.062*	
O1	0.13122 (7)	0.43327 (10)	0.27592 (10)	0.0339 (3)	
O2	0.28543 (7)	0.65724 (9)	0.24881 (10)	0.0350 (3)	
O3	0.33920 (10)	0.54448 (14)	0.42352 (14)	0.0626 (5)	
O4	0.37114 (8)	0.40007 (11)	0.34821 (12)	0.0474 (4)	

### Atomic displacement parameters (Å^2^)

**Table:**

	*U*^11^	*U*^22^	*U*^33^	*U*^12^	*U*^13^	*U*^23^
C1	0.0294 (10)	0.0329 (9)	0.0376 (12)	−0.0013 (7)	−0.0052 (9)	0.0027 (9)
C2	0.0337 (10)	0.0327 (9)	0.0381 (12)	−0.0041 (8)	−0.0040 (9)	0.0019 (9)
C3	0.0464 (12)	0.0394 (11)	0.0460 (15)	0.0039 (9)	−0.0069 (11)	0.0074 (11)
C4	0.0713 (17)	0.0524 (13)	0.0486 (16)	0.0041 (12)	−0.0116 (14)	0.0182 (13)
C5	0.0709 (16)	0.0634 (15)	0.0371 (14)	−0.0021 (13)	0.0034 (12)	0.0091 (13)
C6	0.0453 (13)	0.0455 (12)	0.0418 (14)	−0.0011 (9)	0.0029 (10)	0.0020 (11)
C7	0.0307 (10)	0.0356 (10)	0.0405 (13)	−0.0029 (8)	−0.0040 (9)	0.0040 (10)
C8	0.0299 (9)	0.0324 (9)	0.0371 (12)	−0.0023 (7)	−0.0034 (9)	0.0002 (10)
C9	0.0303 (9)	0.0372 (9)	0.0450 (13)	−0.0005 (7)	0.0022 (10)	0.0097 (10)
C10	0.0358 (12)	0.0577 (13)	0.0527 (16)	−0.0003 (9)	−0.0044 (11)	0.0093 (12)
C11	0.0353 (12)	0.0826 (18)	0.068 (2)	0.0032 (11)	−0.0080 (12)	0.0181 (17)
C12	0.0339 (13)	0.0700 (16)	0.094 (2)	0.0146 (11)	0.0082 (15)	0.0281 (18)
C13	0.0465 (15)	0.0631 (15)	0.112 (3)	0.0139 (11)	0.0137 (16)	−0.0142 (18)
C14	0.0324 (12)	0.0625 (14)	0.083 (2)	0.0039 (10)	−0.0013 (12)	−0.0182 (15)
C15	0.0400 (11)	0.0315 (9)	0.0400 (13)	−0.0053 (8)	0.0004 (9)	0.0015 (9)
C16	0.0499 (12)	0.0406 (11)	0.0561 (16)	0.0033 (9)	−0.0044 (12)	−0.0026 (12)
C17	0.0645 (16)	0.0429 (12)	0.068 (2)	0.0084 (11)	0.0081 (14)	−0.0046 (13)
C18	0.0860 (19)	0.0405 (12)	0.0519 (17)	0.0016 (12)	0.0135 (15)	−0.0092 (12)
C19	0.0799 (18)	0.0442 (12)	0.0452 (16)	−0.0080 (12)	−0.0051 (13)	−0.0051 (12)
C20	0.0567 (14)	0.0352 (10)	0.0447 (14)	−0.0033 (9)	−0.0053 (11)	−0.0012 (11)
C21	0.0290 (10)	0.0312 (9)	0.0355 (12)	0.0008 (7)	−0.0040 (8)	−0.0013 (9)
C22	0.0300 (9)	0.0306 (9)	0.0334 (11)	−0.0006 (7)	−0.0035 (9)	0.0004 (9)
C23	0.0238 (9)	0.0336 (9)	0.0411 (12)	0.0001 (7)	−0.0011 (8)	0.0036 (9)
C24	0.0316 (10)	0.0328 (9)	0.0405 (13)	−0.0010 (7)	0.0000 (9)	0.0017 (9)
C25	0.0314 (10)	0.0399 (10)	0.0431 (14)	0.0018 (8)	−0.0049 (9)	−0.0003 (10)
C26	0.0469 (14)	0.0709 (16)	0.089 (2)	0.0162 (12)	−0.0281 (15)	−0.0003 (17)
C27	0.0543 (16)	0.0868 (19)	0.120 (3)	0.0309 (14)	−0.0158 (18)	0.004 (2)
C28	0.0354 (10)	0.0343 (9)	0.0339 (12)	−0.0073 (8)	0.0003 (9)	0.0066 (10)
C29	0.0539 (14)	0.0430 (11)	0.0385 (13)	−0.0052 (9)	−0.0007 (11)	0.0014 (11)
C30	0.0707 (17)	0.0522 (13)	0.0427 (15)	−0.0120 (12)	−0.0160 (13)	0.0031 (12)
C31	0.0523 (14)	0.0559 (14)	0.0548 (17)	−0.0090 (11)	−0.0204 (12)	0.0107 (13)
C32	0.0385 (11)	0.0414 (10)	0.0496 (15)	−0.0031 (9)	−0.0088 (11)	0.0099 (11)
C33	0.0321 (10)	0.0317 (9)	0.0390 (12)	−0.0050 (7)	−0.0013 (9)	0.0088 (9)
C34	0.0346 (11)	0.0398 (10)	0.0475 (14)	0.0021 (8)	0.0060 (10)	0.0099 (11)
C35	0.0484 (13)	0.0518 (13)	0.0520 (17)	0.0032 (10)	0.0081 (11)	−0.0020 (12)
C36	0.0620 (17)	0.0616 (15)	0.078 (2)	0.0172 (13)	0.0226 (15)	0.0008 (15)
C37	0.0488 (16)	0.0731 (19)	0.152 (4)	0.0151 (14)	0.041 (2)	0.015 (2)
C38	0.0384 (14)	0.0567 (16)	0.195 (5)	−0.0017 (11)	0.027 (2)	0.005 (2)
C39	0.0354 (12)	0.0439 (12)	0.119 (3)	−0.0022 (10)	0.0095 (14)	−0.0018 (15)
C40	0.0273 (9)	0.0345 (9)	0.0459 (14)	−0.0023 (7)	−0.0055 (9)	0.0026 (10)
C41	0.0608 (15)	0.0427 (12)	0.0500 (16)	0.0003 (10)	−0.0029 (12)	−0.0011 (12)
C42	0.0714 (17)	0.0532 (14)	0.0612 (19)	0.0038 (12)	−0.0058 (14)	−0.0173 (14)
C43	0.0637 (15)	0.0354 (12)	0.088 (2)	0.0008 (10)	−0.0147 (16)	−0.0093 (15)
C44	0.0728 (18)	0.0367 (12)	0.084 (2)	−0.0056 (11)	−0.0084 (17)	0.0140 (15)
C45	0.0575 (14)	0.0410 (11)	0.0555 (16)	−0.0058 (9)	0.0024 (12)	0.0048 (12)
O1	0.0302 (7)	0.0346 (7)	0.0368 (9)	−0.0016 (5)	−0.0061 (6)	0.0017 (6)
O2	0.0280 (6)	0.0325 (6)	0.0445 (9)	−0.0022 (5)	−0.0013 (6)	−0.0001 (7)
O3	0.0529 (10)	0.0678 (10)	0.0670 (13)	0.0154 (8)	−0.0274 (9)	−0.0264 (10)
O4	0.0386 (8)	0.0463 (7)	0.0574 (11)	0.0109 (6)	−0.0137 (8)	−0.0016 (8)

### Geometric parameters (Å, °)

**Table:**

C1—O1	1.447 (2)	C23—C40	1.511 (3)
C1—C15	1.507 (3)	C23—C33	1.511 (3)
C1—C2	1.515 (3)	C24—O2	1.442 (2)
C1—C21	1.596 (2)	C24—C34	1.510 (3)
C2—C3	1.374 (3)	C24—C28	1.521 (3)
C2—C7	1.394 (3)	C25—O3	1.190 (3)
C3—C4	1.385 (4)	C25—O4	1.333 (2)
C3—H3	0.9300	C26—O4	1.453 (3)
C4—C5	1.374 (4)	C26—C27	1.482 (4)
C4—H4	0.9300	C26—H26A	0.9700
C5—C6	1.391 (3)	C26—H26B	0.9700
C5—H5	0.9300	C27—H27A	0.9600
C6—C7	1.369 (3)	C27—H27B	0.9600
C6—H6	0.9300	C27—H27C	0.9600
C7—C8	1.525 (3)	C28—C29	1.378 (3)
C8—O1	1.441 (3)	C28—C33	1.387 (3)
C8—C9	1.503 (3)	C29—C30	1.382 (3)
C8—C22	1.553 (2)	C29—H29	0.9300
C9—C14	1.370 (3)	C30—C31	1.385 (4)
C9—C10	1.376 (3)	C30—H30	0.9300
C10—C11	1.391 (3)	C31—C32	1.377 (4)
C10—H10	0.9300	C31—H31	0.9300
C11—C12	1.357 (4)	C32—C33	1.374 (3)
C11—H11	0.9300	C32—H32	0.9300
C12—C13	1.356 (5)	C34—C39	1.378 (3)
C12—H12	0.9300	C34—C35	1.378 (3)
C13—C14	1.384 (3)	C35—C36	1.374 (3)
C13—H13	0.9300	C35—H35	0.9300
C14—H14	0.9300	C36—C37	1.362 (4)
C15—C20	1.387 (3)	C36—H36	0.9300
C15—C16	1.390 (3)	C37—C38	1.363 (5)
C16—C17	1.383 (4)	C37—H37	0.9300
C16—H16	0.9300	C38—C39	1.381 (4)
C17—C18	1.365 (4)	C38—H38	0.9300
C17—H17	0.9300	C39—H39	0.9300
C18—C19	1.369 (4)	C40—C41	1.373 (3)
C18—H18	0.9300	C40—C45	1.380 (3)
C19—C20	1.383 (4)	C41—C42	1.392 (3)
C19—H19	0.9300	C41—H41	0.9300
C20—H20	0.9300	C42—C43	1.354 (4)
C21—C25	1.523 (3)	C42—H42	0.9300
C21—C22	1.561 (3)	C43—C44	1.361 (4)
C21—C24	1.593 (3)	C43—H43	0.9300
C22—C23	1.553 (3)	C44—C45	1.381 (3)
C22—H22	0.9800	C44—H44	0.9300
C23—O2	1.460 (2)	C45—H45	0.9300
			
O1—C1—C15	109.42 (16)	O2—C23—C22	99.75 (13)
O1—C1—C2	100.46 (15)	C40—C23—C22	120.96 (18)
C15—C1—C2	118.86 (16)	C33—C23—C22	109.37 (14)
O1—C1—C21	100.22 (13)	O2—C24—C34	109.60 (15)
C15—C1—C21	119.83 (17)	O2—C24—C28	100.02 (14)
C2—C1—C21	104.83 (17)	C34—C24—C28	117.57 (19)
C3—C2—C7	120.4 (2)	O2—C24—C21	98.09 (15)
C3—C2—C1	134.3 (2)	C34—C24—C21	117.56 (16)
C7—C2—C1	105.33 (17)	C28—C24—C21	110.57 (15)
C2—C3—C4	118.1 (2)	O3—C25—O4	122.92 (19)
C2—C3—H3	121.0	O3—C25—C21	125.05 (18)
C4—C3—H3	121.0	O4—C25—C21	112.00 (18)
C5—C4—C3	121.4 (2)	O4—C26—C27	108.2 (3)
C5—C4—H4	119.3	O4—C26—H26A	110.1
C3—C4—H4	119.3	C27—C26—H26A	110.1
C4—C5—C6	120.8 (3)	O4—C26—H26B	110.1
C4—C5—H5	119.6	C27—C26—H26B	110.1
C6—C5—H5	119.6	H26A—C26—H26B	108.4
C7—C6—C5	117.8 (2)	C26—C27—H27A	109.5
C7—C6—H6	121.1	C26—C27—H27B	109.5
C5—C6—H6	121.1	H27A—C27—H27B	109.5
C6—C7—C2	121.56 (19)	C26—C27—H27C	109.5
C6—C7—C8	133.01 (19)	H27A—C27—H27C	109.5
C2—C7—C8	105.43 (19)	H27B—C27—H27C	109.5
O1—C8—C9	111.80 (17)	C29—C28—C33	119.9 (2)
O1—C8—C7	100.16 (13)	C29—C28—C24	134.64 (19)
C9—C8—C7	115.98 (18)	C33—C28—C24	105.28 (18)
O1—C8—C22	101.58 (16)	C28—C29—C30	118.6 (2)
C9—C8—C22	119.04 (14)	C28—C29—H29	120.7
C7—C8—C22	105.76 (16)	C30—C29—H29	120.7
C14—C9—C10	118.42 (19)	C29—C30—C31	120.9 (2)
C14—C9—C8	120.0 (2)	C29—C30—H30	119.5
C10—C9—C8	121.5 (2)	C31—C30—H30	119.5
C9—C10—C11	120.1 (3)	C32—C31—C30	120.7 (2)
C9—C10—H10	120.0	C32—C31—H31	119.6
C11—C10—H10	120.0	C30—C31—H31	119.6
C12—C11—C10	120.6 (3)	C33—C32—C31	118.1 (2)
C12—C11—H11	119.7	C33—C32—H32	121.0
C10—C11—H11	119.7	C31—C32—H32	121.0
C13—C12—C11	119.6 (2)	C32—C33—C28	121.8 (2)
C13—C12—H12	120.2	C32—C33—C23	132.54 (19)
C11—C12—H12	120.2	C28—C33—C23	105.68 (16)
C12—C13—C14	120.4 (3)	C39—C34—C35	118.7 (2)
C12—C13—H13	119.8	C39—C34—C24	119.9 (2)
C14—C13—H13	119.8	C35—C34—C24	121.36 (19)
C9—C14—C13	120.9 (3)	C36—C35—C34	120.8 (2)
C9—C14—H14	119.6	C36—C35—H35	119.6
C13—C14—H14	119.6	C34—C35—H35	119.6
C20—C15—C16	117.8 (2)	C37—C36—C35	120.4 (3)
C20—C15—C1	120.32 (19)	C37—C36—H36	119.8
C16—C15—C1	121.8 (2)	C35—C36—H36	119.8
C17—C16—C15	120.3 (2)	C36—C37—C38	119.2 (2)
C17—C16—H16	119.9	C36—C37—H37	120.4
C15—C16—H16	119.9	C38—C37—H37	120.4
C18—C17—C16	121.1 (2)	C37—C38—C39	121.2 (3)
C18—C17—H17	119.5	C37—C38—H38	119.4
C16—C17—H17	119.5	C39—C38—H38	119.4
C17—C18—C19	119.5 (2)	C34—C39—C38	119.7 (3)
C17—C18—H18	120.3	C34—C39—H39	120.2
C19—C18—H18	120.3	C38—C39—H39	120.2
C18—C19—C20	120.1 (2)	C41—C40—C45	118.1 (2)
C18—C19—H19	119.9	C41—C40—C23	124.4 (2)
C20—C19—H19	119.9	C45—C40—C23	117.5 (2)
C19—C20—C15	121.2 (2)	C40—C41—C42	120.4 (2)
C19—C20—H20	119.4	C40—C41—H41	119.8
C15—C20—H20	119.4	C42—C41—H41	119.8
C25—C21—C22	109.61 (17)	C43—C42—C41	120.6 (3)
C25—C21—C24	109.38 (15)	C43—C42—H42	119.7
C22—C21—C24	100.92 (14)	C41—C42—H42	119.7
C25—C21—C1	114.84 (16)	C42—C43—C44	119.5 (2)
C22—C21—C1	101.49 (14)	C42—C43—H43	120.2
C24—C21—C1	118.96 (17)	C44—C43—H43	120.2
C8—C22—C23	121.68 (16)	C43—C44—C45	120.5 (3)
C8—C22—C21	101.91 (14)	C43—C44—H44	119.7
C23—C22—C21	102.37 (16)	C45—C44—H44	119.7
C8—C22—H22	110.0	C40—C45—C44	120.7 (3)
C23—C22—H22	110.0	C40—C45—H45	119.6
C21—C22—H22	110.0	C44—C45—H45	119.6
O2—C23—C40	107.06 (14)	C8—O1—C1	99.21 (14)
O2—C23—C33	99.73 (16)	C24—O2—C23	98.41 (13)
C40—C23—C33	116.23 (16)	C25—O4—C26	116.77 (19)
			
O1—C1—C2—C3	148.7 (2)	C1—C21—C24—O2	147.77 (15)
C15—C1—C2—C3	29.5 (3)	C25—C21—C24—C34	39.6 (2)
C21—C1—C2—C3	−107.7 (2)	C22—C21—C24—C34	155.13 (17)
O1—C1—C2—C7	−31.59 (18)	C1—C21—C24—C34	−95.1 (2)
C15—C1—C2—C7	−150.79 (17)	C25—C21—C24—C28	178.55 (16)
C21—C1—C2—C7	72.03 (17)	C22—C21—C24—C28	−65.97 (18)
C7—C2—C3—C4	1.1 (3)	C1—C21—C24—C28	43.8 (2)
C1—C2—C3—C4	−179.2 (2)	C22—C21—C25—O3	−13.6 (3)
C2—C3—C4—C5	−1.2 (4)	C24—C21—C25—O3	96.2 (3)
C3—C4—C5—C6	−0.5 (4)	C1—C21—C25—O3	−127.1 (2)
C4—C5—C6—C7	2.2 (4)	C22—C21—C25—O4	168.36 (16)
C5—C6—C7—C2	−2.3 (3)	C24—C21—C25—O4	−81.9 (2)
C5—C6—C7—C8	178.6 (2)	C1—C21—C25—O4	54.9 (2)
C3—C2—C7—C6	0.7 (3)	O2—C24—C28—C29	143.1 (2)
C1—C2—C7—C6	−179.10 (18)	C34—C24—C28—C29	24.6 (3)
C3—C2—C7—C8	179.99 (18)	C21—C24—C28—C29	−114.3 (3)
C1—C2—C7—C8	0.20 (19)	O2—C24—C28—C33	−32.20 (18)
C6—C7—C8—O1	−149.5 (2)	C34—C24—C28—C33	−150.66 (17)
C2—C7—C8—O1	31.36 (17)	C21—C24—C28—C33	70.45 (18)
C6—C7—C8—C9	−29.0 (3)	C33—C28—C29—C30	0.6 (3)
C2—C7—C8—C9	151.83 (17)	C24—C28—C29—C30	−174.1 (2)
C6—C7—C8—C22	105.3 (3)	C28—C29—C30—C31	−1.9 (3)
C2—C7—C8—C22	−73.85 (19)	C29—C30—C31—C32	0.9 (4)
O1—C8—C9—C14	−173.1 (2)	C30—C31—C32—C33	1.5 (3)
C7—C8—C9—C14	72.9 (3)	C31—C32—C33—C28	−2.9 (3)
C22—C8—C9—C14	−55.1 (3)	C31—C32—C33—C23	174.9 (2)
O1—C8—C9—C10	8.5 (3)	C29—C28—C33—C32	1.8 (3)
C7—C8—C9—C10	−105.4 (2)	C24—C28—C33—C32	177.97 (18)
C22—C8—C9—C10	126.5 (2)	C29—C28—C33—C23	−176.49 (18)
C14—C9—C10—C11	1.4 (4)	C24—C28—C33—C23	−0.36 (19)
C8—C9—C10—C11	179.8 (2)	O2—C23—C33—C32	−145.7 (2)
C9—C10—C11—C12	0.4 (4)	C40—C23—C33—C32	−31.1 (3)
C10—C11—C12—C13	−1.6 (4)	C22—C23—C33—C32	110.2 (2)
C11—C12—C13—C14	0.8 (5)	O2—C23—C33—C28	32.33 (17)
C10—C9—C14—C13	−2.2 (4)	C40—C23—C33—C28	146.94 (16)
C8—C9—C14—C13	179.4 (2)	C22—C23—C33—C28	−71.69 (18)
C12—C13—C14—C9	1.2 (5)	O2—C24—C34—C39	12.0 (3)
O1—C1—C15—C20	6.4 (3)	C28—C24—C34—C39	125.2 (2)
C2—C1—C15—C20	120.9 (2)	C21—C24—C34—C39	−98.7 (3)
C21—C1—C15—C20	−108.4 (2)	O2—C24—C34—C35	−169.2 (2)
O1—C1—C15—C16	−174.34 (18)	C28—C24—C34—C35	−55.9 (3)
C2—C1—C15—C16	−59.9 (3)	C21—C24—C34—C35	80.1 (3)
C21—C1—C15—C16	70.9 (3)	C39—C34—C35—C36	0.0 (4)
C20—C15—C16—C17	3.0 (3)	C24—C34—C35—C36	−178.8 (2)
C1—C15—C16—C17	−176.3 (2)	C34—C35—C36—C37	−1.3 (5)
C15—C16—C17—C18	−0.7 (4)	C35—C36—C37—C38	1.6 (6)
C16—C17—C18—C19	−2.0 (4)	C36—C37—C38—C39	−0.8 (6)
C17—C18—C19—C20	2.2 (4)	C35—C34—C39—C38	0.8 (4)
C18—C19—C20—C15	0.1 (4)	C24—C34—C39—C38	179.7 (3)
C16—C15—C20—C19	−2.7 (3)	C37—C38—C39—C34	−0.5 (6)
C1—C15—C20—C19	176.6 (2)	O2—C23—C40—C41	−109.8 (2)
O1—C1—C21—C25	150.94 (17)	C33—C23—C40—C41	139.8 (2)
C15—C1—C21—C25	−89.5 (2)	C22—C23—C40—C41	3.2 (3)
C2—C1—C21—C25	47.1 (2)	O2—C23—C40—C45	67.2 (2)
O1—C1—C21—C22	32.8 (2)	C33—C23—C40—C45	−43.2 (2)
C15—C1—C21—C22	152.33 (18)	C22—C23—C40—C45	−179.77 (17)
C2—C1—C21—C22	−71.00 (18)	C45—C40—C41—C42	3.1 (3)
O1—C1—C21—C24	−76.68 (19)	C23—C40—C41—C42	−179.9 (2)
C15—C1—C21—C24	42.9 (2)	C40—C41—C42—C43	−2.3 (4)
C2—C1—C21—C24	179.53 (15)	C41—C42—C43—C44	−0.2 (4)
O1—C8—C22—C23	78.17 (19)	C42—C43—C44—C45	1.8 (4)
C9—C8—C22—C23	−45.0 (3)	C41—C40—C45—C44	−1.5 (3)
C7—C8—C22—C23	−177.65 (17)	C23—C40—C45—C44	−178.7 (2)
O1—C8—C22—C21	−34.62 (19)	C43—C44—C45—C40	−1.0 (4)
C9—C8—C22—C21	−157.8 (2)	C9—C8—O1—C1	−174.22 (15)
C7—C8—C22—C21	69.6 (2)	C7—C8—O1—C1	−50.79 (15)
C25—C21—C22—C8	−120.98 (18)	C22—C8—O1—C1	57.78 (16)
C24—C21—C22—C8	123.71 (16)	C15—C1—O1—C8	177.00 (15)
C1—C21—C22—C8	0.9 (2)	C2—C1—O1—C8	51.16 (15)
C25—C21—C22—C23	112.46 (18)	C21—C1—O1—C8	−56.16 (17)
C24—C21—C22—C23	−2.85 (17)	C34—C24—O2—C23	176.01 (18)
C1—C21—C22—C23	−125.70 (16)	C28—C24—O2—C23	51.83 (17)
C8—C22—C23—O2	−145.34 (18)	C21—C24—O2—C23	−60.84 (15)
C21—C22—C23—O2	−32.79 (17)	C40—C23—O2—C24	−173.51 (17)
C8—C22—C23—C40	97.9 (2)	C33—C23—O2—C24	−52.06 (16)
C21—C22—C23—C40	−149.55 (16)	C22—C23—O2—C24	59.70 (17)
C8—C22—C23—C33	−41.3 (2)	O3—C25—O4—C26	−1.5 (3)
C21—C22—C23—C33	71.22 (17)	C21—C25—O4—C26	176.5 (2)
C25—C21—C24—O2	−77.51 (17)	C27—C26—O4—C25	−174.2 (2)
C22—C21—C24—O2	37.98 (16)		

### Hydrogen-bond geometry (Å, °)

**Table:**

Cg8 and Cg10 are the centroids of the C15–C20 and C28–C33 rings, respectively.

**Table:**

*D*—H···*A*	*D*—H	H···*A*	*D*···*A*	*D*—H···*A*
C4—H4···Cg10^i^	0.93	2.97	3.740 (3)	141
C35—H35···Cg8	0.93	2.60	3.446 (2)	151
C44—H44···Cg8^ii^	0.93	2.87	3.671 (3)	145

Symmetry codes: (i) −*x*+1/2, *y*−1/2, *z*+1/2;  (ii) *x*, *y*+1, *z*.
